# Exploring Higher-Order Conceptual Learning in an Arthropod with a Large Multisensory Processing Center

**DOI:** 10.3390/insects13010081

**Published:** 2022-01-12

**Authors:** Kenna D. S. Lehmann, Fiona G. Shogren, Mariah Fallick, James Colton Watts, Daniel Schoenberg, Daniel D. Wiegmann, Verner P. Bingman, Eileen A. Hebets

**Affiliations:** 1 School of Biological Sciences, University of Nebraska-Lincoln, Lincoln, NE 68588, USA; kdslehmann@gmail.com (K.D.S.L.); fionashogren5@gmail.com (F.G.S.); mariah.fallick@unmc.edu (M.F.); dschoenberg@unl.edu (D.S.); 2 Department of Biology, Texas A&M University, College Station, TX 77843, USA; wattsj@goldmail.etsu.edu; 3 Department of Biological Sciences, Bowling Green State University, Bowling Green, OH 43403, USA; ddwiegm@bgsu.edu; 4 J.P. Scott Center for Neuroscience, Mind and Behavior, Bowling Green State University, Bowling Green, OH 43403, USA; vbingma@bgsu.edu; 5 Department of Psychology, Bowling Green State University, Bowling Green, OH 43403, USA

**Keywords:** general intelligence, comparative cognition, amblypygid, associative learning, cross-modal learning

## Abstract

**Simple Summary:**

It is difficult to measure animal intelligence because the definition of ‘intelligence’ varies, and many animals are good at specific tasks used to measure intelligence or cognition. To address this, scientists often look for evidence of common cognitive abilities. One such ability, the ability to learn concepts, is thought to be rare in animals, especially invertebrates. Concepts include the ideas of ‘same’ and ‘different’. These concepts can be applied to anything in the environment while also being independent of those objects and can help animals understand and survive their environment. Amblypygids, a relative of spiders, live in tropical and subtropical areas, are very good learners, and have a large, complex brain region known to process information from multiple senses. We tested whether amblypygids could learn the concept of ‘same’ by training them to move toward a stimulus that matched with an initial stimulus. We also trained some individuals to learn the concept ‘different’ by training them to move toward a non-matching stimulus. When we used new stimuli, the amblypygids did not move toward the correct stimulus significantly more often than the incorrect stimulus, suggesting either they are unable to learn these higher-order concepts or our experimental design failed to elicit that ability.

**Abstract:**

Comparative cognition aims to understand the evolutionary history and current function of cognitive abilities in a variety of species with diverse natural histories. One characteristic often attributed to higher cognitive abilities is higher-order conceptual learning, such as the ability to learn concepts independent of stimuli—e.g., ‘same’ or ‘different’. Conceptual learning has been documented in honeybees and a number of vertebrates. Amblypygids, nocturnal enigmatic arachnids, are good candidates for higher-order learning because they are excellent associational learners, exceptional navigators, and they have large, highly folded mushroom bodies, which are brain regions known to be involved in learning and memory in insects. In Experiment 1, we investigate if the amblypygid *Phrynus marginimaculatus* can learn the concept of same with a delayed odor matching task. In Experiment 2, we test if *Paraphrynus laevifrons* can learn same/different with delayed tactile matching and nonmatching tasks before testing if they can transfer this learning to a novel cross-modal odor stimulus. Our data provide no evidence of conceptual learning in amblypygids, but more solid conclusions will require the use of alternative experimental designs to ensure our negative results are not simply a consequence of the designs we employed.

## 1. Introduction

Cognition and learning are complex behaviors that are difficult to define [1,2,3,4]. This makes levels of cognition and learning difficult to measure and especially difficult to compare among species. Despite this difficulty, comparative cognition is a thriving field tasked with understanding the evolution of complex cognitive functions. This field has succeeded by carefully defining, testing, and comparing specific cognitive abilities of different species. 

A wide variety of assays have been deployed to test the cognitive abilities of animals [3]. Careful manipulation of the caches of food-storing animals demonstrates numerous birds and rodents remember the location of their caches, what was stored there, how long ago it was stored, and whether it was pilfered (reviewed in [5]). In insects, numerous studies using displacements or manipulations of the environment have revealed impressive memory and navigation abilities [6,7]. Extractive foraging problems, wherein an animal must problem-solve to extract a food reward, provide an opportunity to tease apart the many traits that underpin problem solving [8,9], while playback experiments demonstrate a vast array of social information animals perceive and incorporate into their decisions [10,11]. These experiments—predominantly focused on foraging or navigation—demonstrate impressive cognitive abilities, but many of them are domain-specific, meaning that they are not easily applied to new and varied problems. This is in contrast to human cognition, which has been argued to be domain-general ([12] although, see [13] for examples of domain-specific modules in human cognition). 

The specificity or generalizability of learning is of great interest to researchers of animal cognition. While animals that learn can be said to change their behavior or response through experience (although even this definition is fraught, see [14]), some forms of learning, like categorical and associative learning, require learning about common features or the co-occurrence of stimuli. This kind of learning appears widespread in non-human animals. It is presumed to be advantageous, as it can prepare an animal for future tasks, environments, and interactions. However, no amount of categorical or associative learning can prepare an animal for all future contingencies. When faced with such novel contingencies, the ability to apply concepts may prove critical.

The learning of abstract concepts, or higher-order learning, involves an understanding of relationships between stimuli, independent of the stimuli themselves [15]. Abstract concepts can be applied to novel objects and situations, a significant advantage in a dynamic world. Examples of abstract concepts include ‘same’ and ‘different’ or ‘not same’. Such conceptual learning is domain-general, making it easily transferable across contexts. As mentioned previously, domain-general learning is thought to underlie some of humans’ most impressive cognitive abilities (e.g., mathematical concepts and language [16]) and even general intelligence itself [17]. To date, such higher-order learning is thought to be relatively rare in non-human animals, but has been found in a range of vertebrate taxa including goats [18], dolphins [19], sea lions [20], rhesus monkeys [21], echidnas [22], rats [23], and pigeons [24,25]. Birds have received a fair amount of research attention in this area. Pigeons, for example, are able to perceive new stimuli as being within a trained category, such as “car” or “chair” [26], and one experiment went so far as to imprint ducklings on ‘different’ and ‘same’ stimuli. This latter experiment resulted in the ducklings following novel pairs of stimuli so long as they matched the concept they had imprinted on [27].

These studies of higher-order learning have been very informative, but mostly focused on vertebrates. We consider this a grave oversight because invertebrates are relatively easy to study and manipulate in the laboratory, and many studies have demonstrated their wide range of learning abilities [28]. Thus far, invertebrate higher-order learning has been studied predominantly in bees. Honeybees can, for example, learn concepts such as ’same’ and ‘different’; can generalize these concepts across modalities [29]; are successful in ‘oddity’ tasks [30,31]; and demonstrate numerosity [32,33,34]. In association with their extraordinary learning abilities, many hymenopterans are also known for their multimodal integration brain centers, the mushroom bodies (MBs) [35,36]. Indeed, it has been suggested that multimodal MBs are required for conceptual learning (reviewed in [37]). As a group possessing large, complex MBs, members of the nocturnal arachnid group ‘amblypygids’ are excellent candidates for exploring the capacity for conceptual learning.

Amblypygids (Class Arachnida, Order Amblypygi) provide a model system for studying the relationships between sensory systems, neural substrates, and complex behavior such as higher-order learning. The central ganglia of the amblypygid brain is of great interest because of its extensive MBs [35,38]. These MBs are higher brain centers situated in the first segment of arthropod and common ancestor brains [35,36,39,40,41] and are thought to be homologous to the mammalian hippocampus [42]. In insects, MBs have been recognized to power memory, learning, and contextual information synthesis (fruit flies: [43,44,45,46,47,48]; honeybees: [49,50,51,52]; cockroaches: [53,54]). Excitingly, it has been found that amblypygid MBs are immensely more complex in folding compared to their insect counterparts and are multimodal, processing and integrating both visual and olfactory stimuli [38].

Amblypygids also possess prominent sensory organs on their first pair of legs which can successfully detect a multitude of mechanical, substrate-borne, and chemical airborne cues from the surrounding environment [55,56,57,58,59,60,61,62,63,64]. In tandem with these antenniform legs, amblypygids harbor enormous interneurons that facilitate the connection of receptor cells to the central ganglia in the brain. This allows for an unprecedented quickness in the pathway of information in the sensory system ([58] reviewed in [65,66]) and is suggested as being crucial for a multitude of context-specific roles in observed amblypygid behavior (reviewed in [63]). Initial work on amblypygids has focused on their ability to navigate and find their way to a home refuge through complex tropical and subtropical habitats [67,68,69,70]. Due to their impressive navigational abilities, amblypygids have been proffered as a model organism for the study of navigation [71] and recent reviews have summarized the work thus far [72,73].

In addition to the impressive navigational capacities of amblypygids, research has demonstrated that amblypygids are capable of learning and remembering stimuli in multiple sensory modalities. *Phrynus marginemaculatus* learn to discriminate tactile and visual stimuli and can associate specific stimuli in each modality with an available shelter [61,74]. This species has also been shown to integrate multimodal cues into a single representation of a home refuge—i.e., configural learning [75]. *Paraphrynus laevifrons* can navigate to their home shelters on a vertical maze and can return to their previously inhabited shelter based on scent, not location [76]. Self-derived scent is also used by *P. marginemaculatus* to locate a previously used shelter [77], and this species can discriminate between experimenter-presented olfactory cues [78]. Another species, *Damon diadema* behaved differently toward kin vs. non-kin and could locate their mother in a Y-maze via olfaction, suggesting a role of olfaction in recognition [79]. With respect to memory, *P. marginemaculatus* could discriminate scents two weeks after testing [78]. Similarly, in a study of agonistic interactions, individuals of the same species recognized those amblypygids they had interacted with three weeks prior [80].

Considering their sensory physiology, neuroanatomy, and demonstrable cognitive abilities, we hypothesize that amblypygids are capable of employing higher-order conceptual learning. Here we used a delayed match-to-sample task to test the hypothesis that amblypygids can learn the higher-order concepts of ‘same’ and ‘different’. In the first experiment, we tested whether *Phrynus marginemaculatus* could learn to match an initial odor with an open shelter, and then whether they could transfer this learning (i.e., match odor to find open shelter) to novel odors. In the second experiment, we tested whether *Paraphrynus laevifrons* could first learn to associate matched or mismatched (i.e., same/different) tactile cues with an open shelter. We then tested whether they could generalize the concept (i.e., same/different) across modalities to odor cues, effectively testing cross-modal concept transfer. We used two-choice matching and non-matching tests for both experiments. In the training trials, we predicted the likelihood of successful training trials, the proportion of time spent on the correct side, and the proportion of correct first choices would increase with trial number, while the time to enter the correct door would decrease. In test trials, we predicted that test subjects would choose the correct side first more often than expected by chance, and that they would spend more time on the correct side of the test arena.

## 2. Materials and Methods

We housed test subjects in separate plastic cages (22.9 cm × 16.2 cm × 16.5 cm) and attached window screening to the sides, enabling the amblypygids to climb vertically. We kept the room they were housed in at a constant temperature (28 °C) and provided ample humidity with three humidifiers (two Crane Ultrasonic Cool Mist Humidifiers, one Bemis Model 821,000 humidifier). We covered the cage floors with 2.5 cm of coconut coir substrate that we sprayed daily to provide moisture and provided a small petri dish filled with water. Each individual was fed two crickets twice per week.

### 2.1. Experimental Design Overview

To test our two species’ ability to learn to associate stimuli and subsequently transfer that learning to novel stimuli, we used two-choice matching and non-matching tests. Amblypygids exhibit negative phototaxis, thus darkness was used as the trial reward. The training and test trials consisted of presentation of an initial stimulus in a dark start chamber attached to the arena. The initial stimuli differed across the two experiments, with odor cues being used in Experiment 1 and tactile cues in Experiment 2. To begin the assays, the start chamber was flooded with light to encourage the focal individual to enter the test arena. The focal individual was then presented with two doors associated with the two stimuli and leading to darkened reward shelters at opposite ends of the well-lit test arena. During training trials, the correct choice (C+) was presented with an open door to the reward shelter while the incorrect door (C−) was blocked with a barrier. In each test trial, both doors were blocked, and we recorded the test subjects’ first choice and the amount of time spent in front of each door.

### 2.2. Experiment 1—Within Modality “Sameness” Assay

#### 2.2.1. Study Organism

*Phrynus marginemaculatus* Koch, 1840 is a native species to southern Florida (USA), the Bahamas, as well as surrounding Caribbean islands [81,82,83]. They are a wholly nocturnal species that typically inhabits subtropical habitats such as the pine rock hammock in South Florida and Bahamian pine forests [82,83]. These habitats have been likened in structural complexity to tropical rainforests (United States Fish and Wildlife Service 1999). With this in mind, it is expected that the species is capable of fielding complex navigational challenges that demand the neuroanatomy and sensory physiology integral for higher-order learning. Indeed, it is this species that has been the focus of most prior research on learning and navigation [61,75,77]. For this first experiment, we purchased 12 *P. marginemaculatus* from an online source (“Ken the Bug Guy”).

#### 2.2.2. Initial Training Trials

Given that *P. marginemaculatus* is known to be capable of learning odors associated with a shelter [78], we used chemical stimuli paired with putative shelters for our within-modality “sameness” assay. In particular, we initially trained individuals on the two scents used in [78]: geraniol (Sigma-Aldrich, St. Louis, MO, USA, Product Number 163333) and 1-hexanol (Sigma-Aldrich, Product Number 471402). Each individual was run through two training trials/day for five days straight (for ten intial training trials).

**Figure 1:**
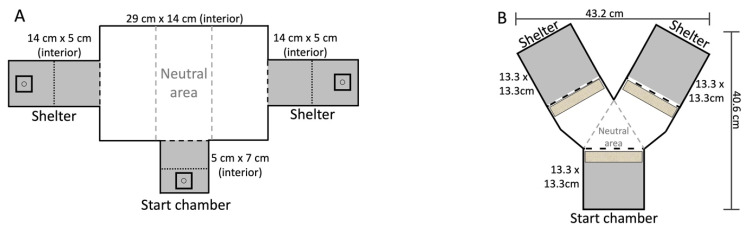
**Training and test trial arenas.** (**A**) Arena for Experiment 1—within modality ‘sameness’ assay. Dotted lines show the configuration of screens used to prevent contact with the stimuli, while black dashed lines represent the shelter openings. The grey dashed lines demonstrate how the arena was split into thirds to calculate the amount of time the amblypygid spent associating with the C+ and C− stimulus. (**B**) Arena for Experiment 2—Cross modal same/different assay. Same as A, except tan shaded areas represent where stimuli were placed surrounding the doors.

We used an identical training/testing arena as was used in prior amblypygid learning experiments (see [78]). Briefly, the rectangular arena was made of opaque white acrylic with three rectangular slots (3 cm × 1 cm) at floor level. Each of the two end slots could open to a reward shelter, while one slot centered in the middle of the long arena side opened into a starting shelter (Figure 1A). All three shelters were constructed out of black acrylic plastic. The two end shelters and starting chamber were divided by a plastic mesh screen into two equal-sized regions. In the regions farthest from the shelter entrance, and inaccessible to the amblypygid due to a mesh screen, we placed a (2″ × 2″) clear plastic tray. A 1.5″ × ⅜″ cotton roll (3D Dental, Euclid, OH, USA) was cut into thirds and placed in the holding trays within each shelter. The assigned odor was then pipetted into the cotton, saturating it.

To train amblypygids to associate a familiar odor with an open shelter, we first exposed them to a pre-assigned odor in their starting shelter. Once animals were released into the main arena, the shelter associated with the matched odor was open, while the other shelter door was blocked off. In particular, during each trial, the cotton wicks were saturated with 10 μL of geraniol or 1-hexanol. The C+ (conditioned stimulus) odor was positioned in the start box in the same manner as the open shelter to act as an initial stimulus. The C− odor was placed in the closed shelter with the same cotton and tray method. In both shelters and the start box, the amblypygid was blocked with mesh from coming in direct contact with the odor tray/saturated cotton. This ensured that no scent was directly on the amblypygid’s legs or body throughout the trials.

To begin a trial, an amblypygid was placed in the darkened start chamber for a five-minute acclimation period. After this acclimation period, the opaque lid was removed to flood the start shelter with light. If the subject did not leave the start chamber after 15 min, it was gently coaxed with a blunt dissection probe into the neutral area (N) in the center of the arena. After exiting the start chamber, the entrance was blocked behind them so they could not re-enter (Video S1).

Training trials lasted for 20 min. Upon entering the C+ shelter, the amblypygid was rewarded with five minutes in the dark before it was removed and returned to its home cage. If the individual did not enter the C+ shelter after 20 min, the learning trial was noted as unsuccessful. Nonetheless, the individual was guided to enter the C+ shelter and allowed to remain in the shelter for the reward period. We switched the side of the C+ shelter at random by flipping a coin for each learning trial. This ensured that our results would not be influenced by potential side preferences exhibited in amblypygids.

Two training trials were conducted for each amblypygid, each day, for 5 consecutive days (a total of 10 training trials). The C+ odor for the first trial of each day was determined at random by flipping a coin. The second training trials switched the C+ odor—i.e., day 1, training 1, C+ = geraniol; day 1, training 2, C+ = hexanol. This was meant to ensure that amblypygids were learning the concept of same and not a specific odor.

During all training trials, we recorded (i) whether the amblypygid entered within the 20-min time limit (success Y/N); (ii) how long it took the amblypygid to enter the shelter associated with the correct stimulus (time to success); (iii) how long the amblypygid spent in each area of the arena (C+/N/C−); and (iv) which side of the arena they entered first (first choice).

To score association times, we separated the central arena into equal thirds: C+ being the side of the arena that contained the matched odor, neutral (N) being the center portion of the arena adjacent to the start box, and C− being the side with the shelter containing the unmatched odor. The areas were defined by marking a (8.5″ × 11″) sheet of paper with lines that denoted the arena in three equal areas. This was placed under the clear bottom of the arena in a way that made it visible while live-scoring and video scoring. All training and test trials were video recorded and live scored.

The arena and shelters were all cleaned and wiped thoroughly with a diluted ethyl acetate solution between each trial to remove any lingering scents.

#### 2.2.3. Trained Odor Test

After the five-day training period was completed, on the 6th day, each amblypygid was run through a test trial with the same chemical stimuli used during training. The first test trials were identical to all training trials except that both shelter openings were blocked so that the amblypygid had no access to either refuge. We recorded (i) which side (C+/C−) the amblypygid entered first from the neutral zone (N) and (ii) how long the amblypygid spent in each of the three arena areas (C+/N/C−).

#### 2.2.4. Reinforcement Training Trials

Before testing whether the amblypygids had learned the concept of same, we conducted another two days of two training reinforcement trials, for a total of four. These trials followed the same protocol as all the previous training trials with the same odors (geraniol, 1-hexanol).

#### 2.2.5. Novel Odor Test

The day after completion of the two reinforcement training days, individuals underwent a Novel Odor Test with two novel odors, 1-octanol (Sigma-Aldrich, Product Number 472328) and hydroxycitronellal (Sigma-Aldrich, Product Number W258318). This allowed us to test if individuals could cognitively apply the principle of sameness learned in training to new conditions. Similar to our Trained Odor Tests, C+ odors were selected at random. Both shelters were again closed, and we scored these trials the same as the Trained Odor Test.

### 2.3. Experiment 2—Cross-Modal Same/Different Assay

#### 2.3.1. Study Organism

For the test of learning ‘same’ and ‘different’, we used *Paraphrynus laevifrons* Pocock, 1894. This species of amblypygid is native to the tropical rainforests of Costa Rica and are most often found along creeks and in areas with numerous potential refuges, ample vertical surface area, and below a sheltering overhang [84]. We opportunistically used this species, as it was already present in the laboratory for other research purposes [85]. We could not use the same experiment design, however, as *P. laevifrons* tends to inhabit vertical, as opposed to horizontal, surfaces [86]. Thus, we designed and built a novel test arena that allowed for vertical climbing associated with shelter entrances. Additionally, the initial training stimuli for *P. laevifrons* were tactile, not odor cues, because we were additionally interested in exploring whether this species was capable of tactile learning. We note that tactile learning has already been demonstrated in *Phrynus marginemaculatus* [61]. Finally, we increased the complexity of this experiment by adding a conceptual learning treatment of ‘different’ and by adding a cross-modal test trial.

#### 2.3.2. Training Trials

As in Experiment 1, *P. laevifrons* were trained to solve a two-choice test by navigating from a start chamber with an initial stimulus to the C+ side of the area, where they would find an open door leading to a darkened chamber. To test whether *P. laevifrons* could apply the concepts of ‘same’ and ‘different’, we divided the ten test subjects into two groups of five. The ‘same’ treatment group was given a two-choice matching test and trained to expect the initial stimulus to be associated with the open door. The ‘different’ treatment group was given a two-choice non-matching test and trained to expect the initial stimulus to be associated with a closed door while a second ‘correct’ stimulus was associated with the open door.

The clear acrylic arena for this experiment was a modified version of a Y-maze, with the start chamber at the base of the Y and the two reward shelters at either end of the Y arms (Figure 1B). The reward shelter and start shelter were darkened by attaching foil to their outsides and lids. As in Experiment 1, the arena and start chamber had clear lids to allow light to flood the arena and provide a negative stimulus during each trial. The door leading from the start chamber to the arena and the doors leading from the arena to the reward shelters were surrounded with different grades of sandpaper to provide the tactile stimulus. Specifically, we used black, heat-treated, waterproof aluminum oxide abrasive (Norton Abrasives, Worcester, MA, USA) in 80 coarse grit and 320 extra fine grit. These grit grades are similar to grades shown to be distinguishable by *P. marginemaculatus* [61,75].

The arena was separated into three equal areas, a neutral area (N) directly in front of the start chamber door, a C− area in front of the door with the non-target stimulus, and a C+ area in front of the door with the target stimulus and an open door leading to the darkened reward chamber. To allow vertical climbing, these doors (3 cm × 1 cm) were positioned 3 cm high. Once again, the initial stimulus and the C+ side of the Y-maze were alternated among trials to prevent the amblypygids from learning a single stimulus response and to correct for any left/right side bias.

To start a trial, an amblypygid was placed in the darkened initial chamber for four minutes to acclimate and experience the tactile stimulus. After this period, the opaque top was removed and the door to the arena was opened. If the subject did not enter the arena of its own accord, it was encouraged to move with a blunt probe tool and the door was closed once it fully entered the test arena. The amblypygid was given fifteen minutes to explore the test arena and move through the open door associated with the C+ stimulus and leading to the reward shelter. After 15 min, individuals were encouraged to enter the reward shelter with a blunt probe where they were given four minutes to rest before the next trial (Video S2). Each subject received four training trials each day for three days. At the end of each day, a fifth test trial was conducted. For each training trial, we recorded (i) latency to enter the reward shelter (time to success) and (ii) whether the subject entered the C+ shelter within 15 min (success Y/N). For this species and test, we did not record first choice because it was uninformative. *Paraphrynus laevifrons* is much larger than *P. marginemaculatus* and the test chamber in this experiment was smaller than the one used for Experiment 1. This often resulted in *P. laevifrons* either touching both stimuli with its long antenniform legs without leaving the center area or entering one side of the arena immediately upon exiting the start chamber. Further, the tactile cue used in this experiment required the individual to touch each stimulus, while the olfactory cues in Experiment 1 could be sampled from the air within the center arena without requiring movement from the individual.

#### 2.3.3. Test Trials

Test trials were identical to training trials except both doors (C+/C−) remained closed throughout the trial. During the first two days of training trials, we ended the day with a test trial using the training tactile stimuli. On the third day, the final test trial was conducted with novel stimuli. This final test examined the subject’s ability to apply the ‘same’ and ‘different’ concepts across modalities by using two scents as the stimuli instead of tactile cues. We used the following two scents as our test stimuli—lavender (Young Living Essential Oils, Lehi, UT 84043, USA) and lemongrass (NOW Foods, Bloomingdale, IL 60108, USA). These novel olfactory stimuli were applied to filter paper and adhered to the plastic wall of the chamber in a fashion identical to the tactile stimuli. For each test trial (days 1–3), we recorded the first door to be ‘probed’, a behavior where the amblypygid repeatedly taps the door and the wall around the door with its antenniform legs (first choice), and calculated the time spent in each of the three arena areas (C+/N/C−).

### 2.4. Statistical Analyses

We used four tests to determine if amblypygids were improving over the course of the training trials. First, for Experiment 1 only, we used a generalized binomial mixed model to model the likelihood of a correct first choice (Y/N), with subject identity as a random effect to control for variation in learning among subjects. The first choice was the first side of the arena that was entered from the neutral zone. Second, for both experiments, we modeled likelihood of success with a generalized binomial mixed model with subject identity as a random effect. Success (Y/N) was defined as entering the shelter associated with the correct stimulus through the only open door of the arena within the training trial time limit (20 min for Experiment 1, 15 min for Experiment 2). We compared this model with and without the trial number as a fixed effect to determine if trial number increased model fit or predicted the likelihood of success. Third, we used a generalized linear mixed model with a beta distribution to model the proportion of time spent on the correct side of the arena. This was only done for Experiment 1 because these data were not collected during the training trials of Experiment 2. We included subject identity as a random effect and compared model fit with and without trial number to determine if amblypygids spent a higher proportion of their time on the correct side of the arena as training progressed. Fourth, for both experiments, we used the training trials to fit a Cox proportional-hazards model and compared this model with and without the trial number as a fixed effect to determine if trial number increased model fit.

We tested the prediction that amblypygids would choose the correct side during test trials more often than expected by chance (50% correct, 50% incorrect) with a Chi-square goodness of fit. We used the Bonferroni correction [87] to account for multiple tests, resulting in an alpha equal to 0.025 and 0.01 for experiment 1 and experiment 2, respectively. We also compared the amount of time spent in each area of the arena (C+, N, and C−) and modeled the proportion of time spent in the correct versus incorrect areas using a beta regression model.

Models were compared via AIC and used betareg [88], lme4 [89], glmmTMB [90], and survival [91] for the beta regression model, generalized binomial mixed model, generalized linear mixed model with beta distribution, and Cox proportional-hazards models, respectively. Figures were created with ggplot2 [92], and figure layouts were created with cowplot [93]. All analyses and figures were done in R (4.1.1) [94] and RStudio (2021.09.1) [95] with the help of tidyverse [96].

## 3. Results

### 3.1. Experiment 1—Within Modality ‘Sameness’ Assay

**Figure 2:**
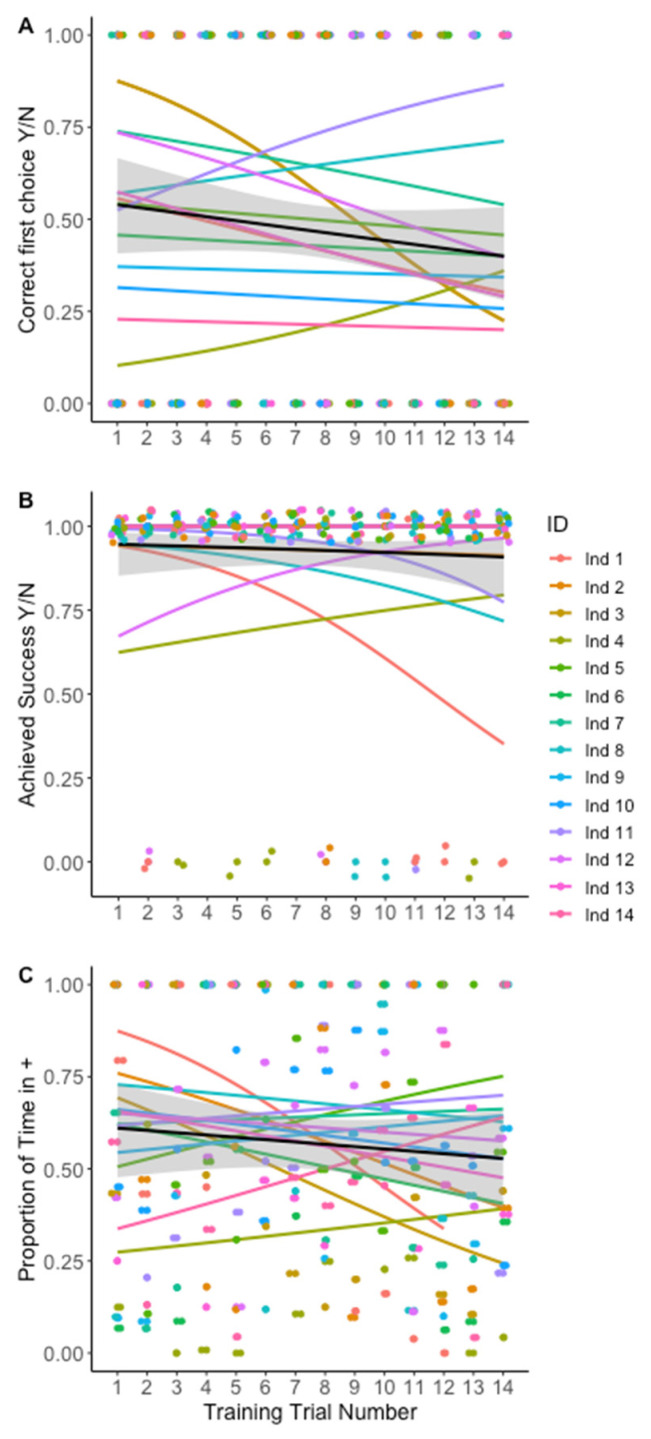
**Measurements of training trial success for Experiment 1—Within modality ‘sameness’ assay.** (**A**) Likelihood of the focal amblypygid first entering into the C+ region from the neutral area. Individuals did not improve their first choice performance during training. (**B**) Likelihood of entering the C+ door in the 20 min time limit (successful training trial). Individuals did not improve in proportion of successful trials over time. (**C**) Proportion of time spent in C+ area out of total time spent outside the neutral zone. A value of 0.5 indicates equal time in the C+ and C− areas of the arena. Individuals did not increase the proportion of time spent associating with the C+ stimulus.

**Figure 3:**
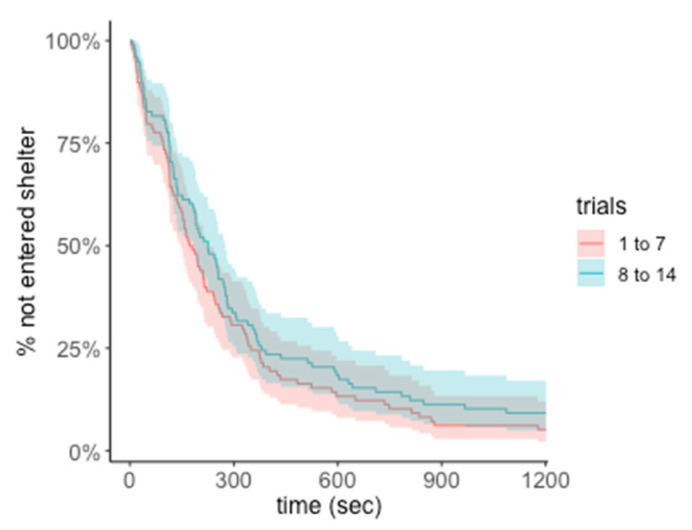
**Cox proportional-hazards model of time to success for Experiment 1—Within modality ‘sameness’ assay.** Survival curves for time to enter correct shelter with 95% confidence intervals. Curves represent early trials (1 to 7) and late trials (8 to 14). Trials were completed at 1200 s.

In our analysis of the training trials, we did not find clear evidence of improvement over time. The likelihood of a correct first choice and the likelihood of success did not increase with trial number. *Phrynus marginemaculatus* did, however, enter the C+ shelter without encouragement within the time limit in the majority of trials (182 of 196 trials, 92.9%). The proportion of time on the correct side also did not increase with trial number, and the time to success did not decrease with trial number as predicted (Figure 2A–C). In all of these models, the trial number did not improve model fit or significantly predict the response variable (Table 1). In the Cox proportional-hazards model, trial number was not a significant predictor of time to enter the correct door (Figure 3, TrialNo coef = −0.019, SE = 0.019, *p* = 0.301)

**Figure 4:**
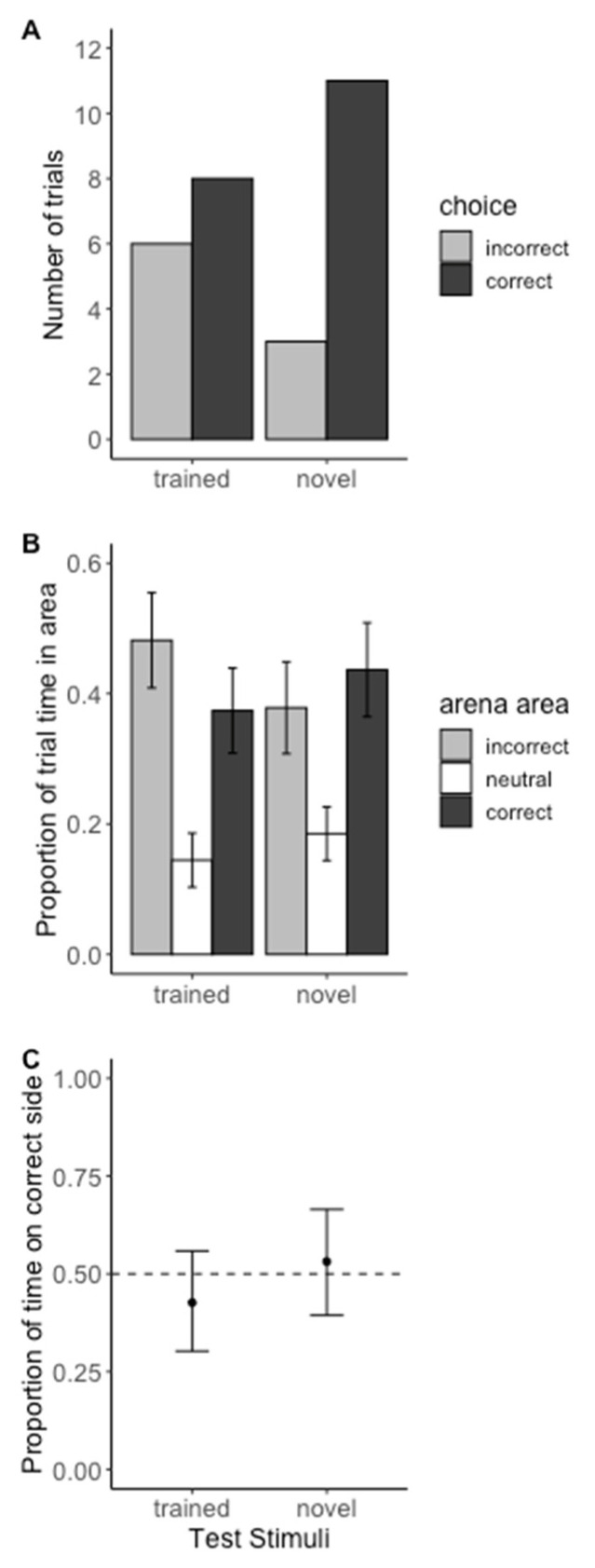
**Test trial results from Experiment 1—within modality ‘sameness’ assay**. (**A**) Barplot of initial choice in two-choice tests. The initial choice for trained stimuli tests (X-squared = 0.28571, df = 1, *p*-value = 0.593) and the initial choice for novel stimuli tests (X-squared = 4.5714, df = 1, *p*-value = 0.03251) were not statistically different. (**B**) Average proportion of time spent in each area of the arena during test trials with standard error bars. (**C**) Modeled estimates and their 95% confidence intervals of the proportion of time spent in front of C+ door (excludes neutral zone). A value of 0.5 represents an equal amount of time spent in front of both doors. *Phrynus marginemaculatus* did not spend significantly more time in front of the C+ door, suggesting they did not learn the concept of ‘same’.

*Phrynus marginemaculatus* did not chose the correct side of the area more often than the incorrect side when presented with the olfactory stimuli that were present during the training trials (Chi-squared = 0.29, *p* = 0.59), or when presented with novel scents (Chi-squared = 4.57, *p* = 0.032). Note, however, that our corrected *p*-value cutoff for this experiment was *p* < 0.025, and thus this result is nearing statistical significance. Nonetheless, these results do not support our hypothesis that *P. marginemaculatus* is capable of learning higher-order concepts such as ‘same’ (Figure 4A). We also did not find support for our prediction that *P. marginemaculatus* would spend a higher proportion of time on the correct side of the arena during test trials (Figure 4B,C, trained mean = 0.43, 95% CI = (0.30, 0.56); novel mean = 0.53, 95% CI = (0.39, 0.66)).

### 3.2. Experiment 2—Cross-Modal Same/Different Assay

**Figure 5:**
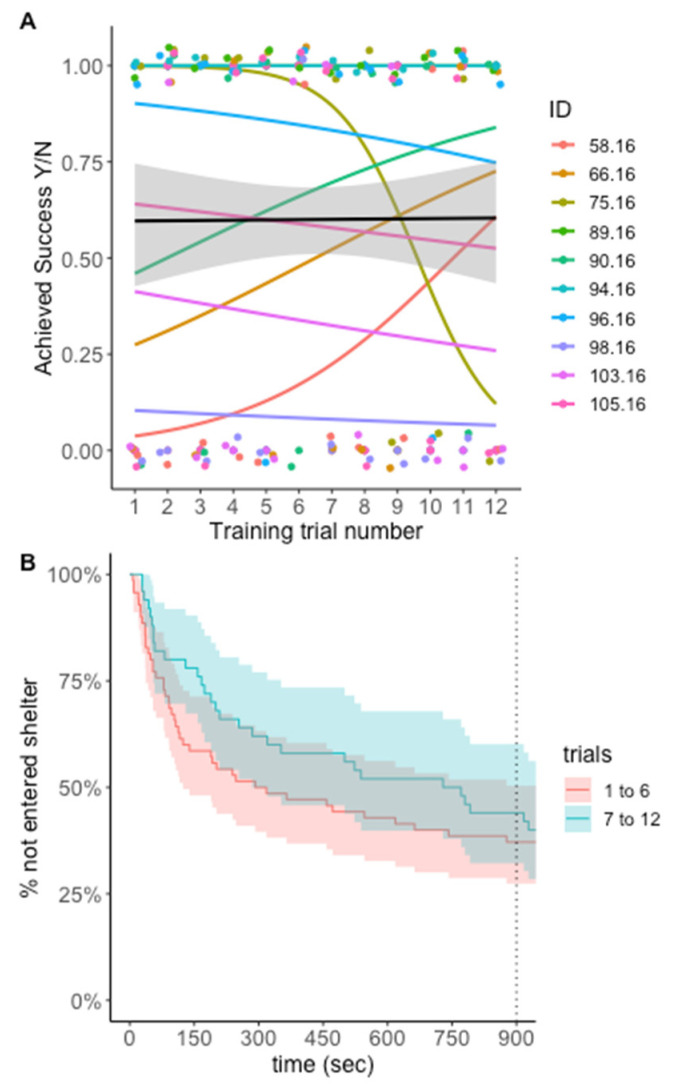
**Measurements of training trial success for Experiment 2—Cross-modal same/different assay.** (**A**) Likelihood of entering the C+ door in the 15 min time limit (successful training trial). Individuals did not improve in proportion of successful trials over time. (**B**) Survival curves for time to enter correct shelter with 95% confidence intervals. Curves represent early trials (1 to 6) and late trials (7 to 12). Dotted line indicates trial end time.

*Paraphrynus laevifrons* were not more likely to succeed or to decrease their time to success in training trials over time (Figure 5). In the model of likelihood of success, the trial number did not improve model fit or significantly predict likelihood of success (Table 2), but success was achieved in a majority of training trials (72 of 120 trials, 60%). In the Cox proportional-hazards model, trial number was not a significant predictor of time to enter the correct door (TrialNo coef = −0.013, SE = 0.033, *p* = 0.701)

**Figure 6:**
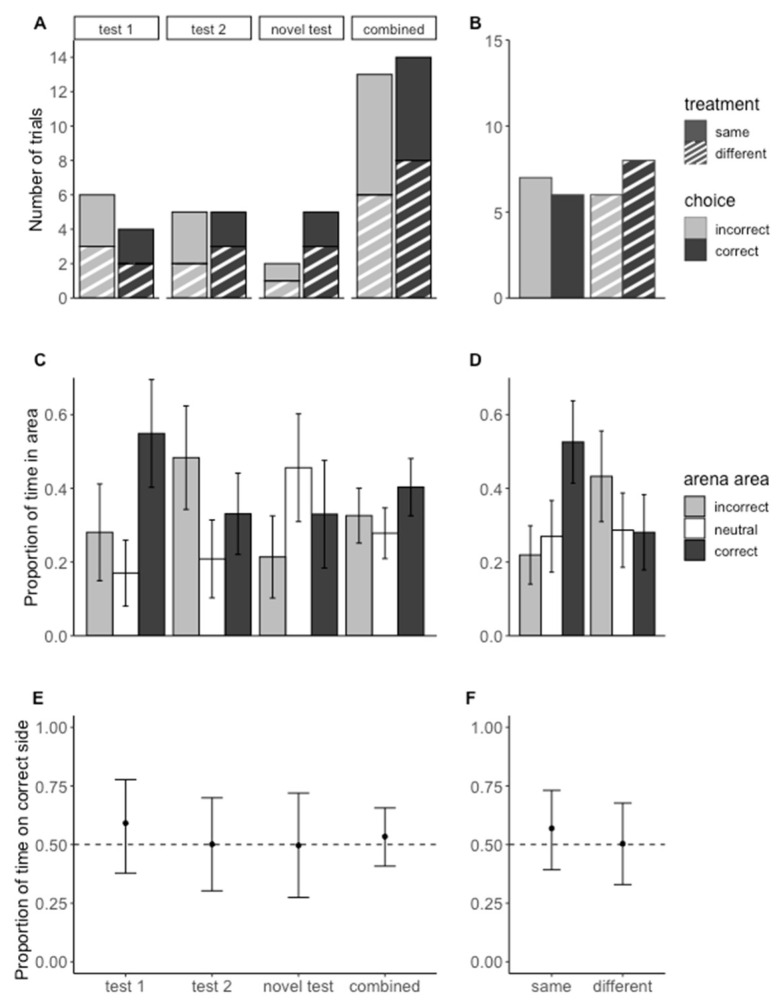
**Test trial results from Experiment 2—Cross-modal same/different assay**. (**A**) Barplot of initial choice in two-choice tests. The initial choice for trained stimuli tests was not significantly different on test day 1 (X-squared = 0.4, *p*-value = 0.53), test day 2 (X-squared = 0, *p*-value = 1), or for the novel stimulus tests on day 3 (X-squared = 1.29, *p*-value = 0.26). Three trials were dropped from the analysis of the novel test because the amblypygids did not probe either the C+ or C− doors. The ‘combined’ bars are the results from all three days of testing combined. (**B**) Barplot of initial choice in two-choice tests for individuals trained to go to the same stimulus as the initial and those trained to go to the different stimulus combined from all three testing days. The same and different treatment groups’ choices were not statistically different from chance (same: X-squared = 0.08, *p*-value = 0.78; different: X-squared = 0.29, *p*-value = 0.59). Two trials were dropped from the ‘same’ group and one from the ‘different’ group because the amblypygids did not probe either door. (**C**) Average proportion of time spent in each area of the arena during test trials for all days of testing and all days combined with standard error bars. (**D**) Average proportion of time spent in each area of the arena during test trials where the individuals were trained to learn ‘same’ and ‘different’ with standard error bars (**E**,**F**). Modeled estimates and their 95% confidence intervals of the proportion of time spent in front of C+ door (excludes neutral zone). A value of 0.5 represents an equal amount of time spent in front of both doors. Individuals did not spend significantly more time in front of the C+ door, indicating *P. laevifrons* did not learn the concepts of ‘same’ and ‘different’.

Three trials were dropped from the analysis of the novel test (2 from the same treatment and 1 from the different treatment) because the amblypygid did not probe either the C+ or C− doors. *Paraphrynus laevifrons* did not choose the correct side of the arena more often than the incorrect side on day 1 or day 2 of test trials when presented with the tactile stimuli with which they had been trained (Test 1: Chi-squared = 0.40, *p* = 0.53; Test 2: Chi-squared = 0, *p* = 1) or when presented with a novel, cross-modal olfactory stimulus (Chi-squared = 1.29, *p* = 0.26). *Paraphrynus laevifrons* did not choose the correct stimulus across all three days of test trials when trained to the matching (Chi-squared = 0.08, *p* = 0.78) or non-matching stimuli (Chi-squared = 0.29, *p* = 0.59, Figure 6A,B).

The test subjects did not spend more time on the correct side of the arena versus the incorrect side on average (Figure 6C,D) (Figure 6E,F; test 1 mean = 0.59, 95% CI = (0.38, 0.78); test 2 mean = 0.51, 95% CI = (0.30, 0.70)); novel test mean = 0.50, 95% CI = (0.27, 0.72); combined mean = 0.53, 95% CI = (0.41, 0.66); same mean = 0.57, 95% CI = (0.39, 0.73); different mean = 0.50, 95% CI = (0.33, 0.68).

## 4. Discussion

Overall, given our experimental design, we found no evidence that amblypygids are capable of higher-order learning of the concepts of same and different. Despite an absence of convincing evidence that *P. marginemacultus* and *P. laevifrons* are capable of abstract associations, we nonetheless view these experiments and associated data as intriguing patterns warranting future study. Indeed, we think it is possible that these findings are the result of inadequate training protocols [15] rather than an inability of amblypygids to exhibit conceptual learning. Unfortunately, so little is known about the natural history of these animals [81] that effective laboratory-based experimental designs remain the largest challenge to assessing their cognitive capacities.

In both experiments, we did not see clear evidence of improvement over the course of learning trials. This, combined with ample evidence of associational learning in *P. marginemaculatus* [74,75,77,78,97], suggests that either our training trials or the period of training were not sufficient for *P. marginemaculaus* and *P. laevifrons* to learn the intended associations in our experiments. The trends toward significance in the novel stimulus trials in both experiments may reflect that these novel trials were conducted after more training trials than the trained stimulus trials. In addition, previous experiments of associational learning in amblypygids have included training trials that lasted anywhere from an hour [61] to an entire 12-h day or night period [75,76,77], while our training exposures were limited to 20 and 15 min in Experiment 1 and 2, respectively. In addition, we switched the C+ stimulus between each training trial, increasing the difficulty of the learning task. While this is key to demonstrate conceptual learning at a level above associational learning, future designs might be more successful if stimuli are consistent for a few trials in a row before switching or if more stimuli are used [24].

In the majority of training trials, *P. marginemaculatus* and *P. laevifrons* entered the C+ shelter within the trial time limit without requiring guidance. This suggests the amblypygids were aware of the shelter location but were insufficiently motivated to enter. It is entirely possible that the bright lights were not a sufficiently negative stimulus, especially since our experiments were run during the animal’s normal daylight schedule. Additionally, observations of amblypygids in novel environments suggest an inquisitive animal, as they tend to engage in apparent exploration following an initial motionless period. Our observed lack of a decrease in time to enter the shelter may, in fact, reflect the amblypygid’s exploratory behavior. Future experiments should be conducted during the amblypygids’ dark period, when they may be more active and more aversive to light. Additional negative stimuli, such as heat, could also be explored to encourage shelter-seeking behavior (e.g., [98]). In future experiments, we plan to use novel scents that differ more from the original trained scents to ensure that our transfer trials demonstrate true concept transfer from trained to novel stimuli.

We also find it important to note that many of the previously discussed learning experiments in other taxa require test subjects to exhibit a threshold level of learning during training trials before proceeding to subsequent test trials [29]. Unfortunately, given our small sample sizes, we were unable to disqualify any individuals from the test trials. We did, however, observe a large amount of individual variation (Figure 2 and Figure 5) that could be due a range of factors including variation in motivation to seek shelter, variation in exploratory behavior, or variation in learning ability itself (as seen in goats [18]). Without a larger sample size, we are unable to determine which of these factors was likely responsible for the individual-level variation we encountered, but future studies should explore these differences.

Given the lack of improvement over the course of training trials, it is not surprising that we did not see evidence of conceptual learning in amblypygids. Their learning, however, may not be expressed in the ways we might expect—e.g., not emerging until confronted with a novel stimulus. For example, in both experiments, we see intriguing trends to suggest that novel stimuli may reveal the amblypygids’ learning abilities. While not a significant difference, the amblypygids chose the C+ door more often than the C− door in the novel tests in both experiments. If our amblypygids are aware of the C+ shelter location but feel no need to enter it, the novel stimulus may present enough uncertainty for the amblypygid to immediately check the expected location of the open door. We look forward to modifications of these experiments, perhaps with a longer training period, more training trials, additional sources of motivation, and a larger sample size to better test these possibilities.

Of course, it is entirely possible that amblypygids are not capable of higher-order learning of concepts such as same/different. Some species have been unable to learn match-to-sample tests, including archerfish [99,100] and cichlids [101]. Amblypygids may not require higher-order concepts to navigate and survive, and higher-order learning may be more costly than associative learning. What differences between amblypygids and bees may account for the difference in their abilities? Both bees and amblypygids are central place foragers, but bees have a more specialized and stationary diet. Perhaps bees lean on the same/different concept to identify new food sources, while amblypygids are opportunistic generalists that must make rapid, undiscerning foraging decisions. Honeybees are eusocial while amblypygids have few social interactions [81]. Does sociality somehow require conceptual learning? These questions can only be answered with a comparative approach, which requires additional experiments on higher-order learning in amblypygids and a wide range of other arthropods.

In summary, we found no concrete evidence of higher-order learning in two species of amblypygid, an arachnid order that is known for its navigational abilities and large, complex mushroom bodies. Future experiments should expand on this work with a larger sample size and modified test conditions to further evaluate whether amblypygids are capable of conceptual learning.

## Figures and Tables

**Table 1:** **Model results for Experiment 1—within modality ‘sameness’ assay.** All models were created with behavioral data from training trials. ∆ AIC is the difference between the AIC of the model with and without trial number as a predictor. Positive ∆ AIC indicates the model shown had a higher AIC value and is a worse model fit than the model without trial number. Including trial number in these models did not significantly improve model fit, indicating *P. marginemaculatus* did not improve over training trials.

Model Response Variable	Predictors	Log-Odds/Estimates	SE	*p*-Value
Correct First Choice (Y/N)	(Intercept)	0.20	0.32	0.525
	TrialNo	−0.04	0.04	0.220
	Observations	196	∆AIC	0.49
Success (Y/N)	(Intercept)	3.68	0.93	<0.001
	TrialNo	−0.05	0.07	0.494
	Observations	196	∆AIC	1.53
Proportion of Time Spent on Correct Side	(Intercept)	0.71	0.20	<0.001
	TrialNo	−0.03	0.02	0.186
	Observations	195	∆AIC	−0.25

**Table 2:** **Model results for Experiment 2—Cross-modal same/different assay.** All models were created with behavioral data from training trials. ∆AIC is the difference between the AIC of the model with and without trial number as a predictor. Positive ∆AIC indicates the model shown had a higher AIC value and is a worse model fit than the model without trial number. Including trial number in this model did not significantly improve model fit, indicating *P. laevifrons* did not improve likelihood of success over training trials.

Model Response Variable	Predictors	Log-Odds/Estimates	SE	*p*-Value
Success (Y/N)	(Intercept)	0.62	0.71	0.384
	TrialNo	0.00	0.06	0.947
	Observations	120	∆AIC	1.99

## Data Availability

The data and code for this study are openly available in github at https://github.com/kdslehmann/AmblypygidCognition_Insects.git (accessed 23 November 2021).

## Supplementary Materials

The following are available online at https://www.mdpi.com/article/10.3390/insects13010081/s1, Video S1: *Phrynus marginimaculatus* completing a training trial for Experiment 1. Video S2: *Paraphrynus laevifrons* completing a training trial for Experiment 2.
